# Sparsentan ameliorates glomerular hypercellularity and inflammatory-gene networks induced by IgA1-IgG immune complexes in a mouse model of IgA nephropathy

**DOI:** 10.1152/ajprenal.00253.2023

**Published:** 2024-03-21

**Authors:** Colin Reily, Zina Moldoveanu, Tiziano Pramparo, Stacy Hall, Zhi-Qiang Huang, Terri Rice, Lea Novak, Radko Komers, Celia P. Jenkinson, Jan Novak

**Affiliations:** ^1^Department of Medicine, University of Alabama at Birmingham, Birmingham, Alabama, United States; ^2^Department of Microbiology, University of Alabama at Birmingham, Birmingham, Alabama, United States; ^3^Travere Therapeutics Incorporated, San Diego, California, United States

**Keywords:** angiotensin, gene expression, glomerulus, IgA nephropathy, inflammation

## Abstract

IgA nephropathy (IgAN) is characterized by glomerular deposition of immune complexes (ICs) consisting of IgA1 with *O*-glycans deficient in galactose (Gd-IgA1) and Gd-IgA1-specific IgG autoantibodies. These ICs induce kidney injury, and in the absence of disease-specific therapy, up to 40% of patients with IgAN progress to kidney failure. IgA1 with its clustered *O*-glycans is unique to humans, which hampered development of small-animal models of IgAN. Here, we used a model wherein engineered ICs (EICs) formed from human Gd-IgA1 and recombinant human IgG autoantibody are injected into nude mice to induce glomerular injury mimicking human IgAN. In this model, we assessed the protective effects of sparsentan, a single-molecule dual endothelin angiotensin receptor antagonist (DEARA) versus vehicle on EIC-induced glomerular proliferation and dysregulation of gene expression in the kidney. Oral administration of sparsentan (60 or 120 mg/kg daily) to mice intravenously injected with EIC attenuated the EIC-induced glomerular hypercellularity. Furthermore, analysis of changes in the whole kidney transcriptome revealed that key inflammatory and proliferative biological genes and pathways that are upregulated in this EIC model of IgAN were markedly reduced by sparsentan, including complement genes, integrin components, members of the mitogen-activated protein kinase family, and Fc receptor elements. Partial overlap between mouse and human differentially expressed genes in IgAN further supported the translational aspect of the immune and inflammatory components from our transcriptional findings. In conclusion, our data indicate that in the mouse model of IgAN, sparsentan targets immune and inflammatory processes leading to protection from mesangial hypercellularity.

**NEW & NOTEWORTHY** The mechanisms by which deposited IgA1 immune complexes cause kidney injury during early phases of IgA nephropathy are poorly understood. We used an animal model we recently developed that involves IgA1-IgG immune complex injections and determined pathways related to the induced mesangioproliferative changes. Treatment with sparsentan, a dual inhibitor of endothelin type A and angiotensin II type 1 receptors, ameliorated the induced mesangioproliferative changes and the associated alterations in the expression of inflammatory genes and networks.

## Graphical abstract

**Figure:**
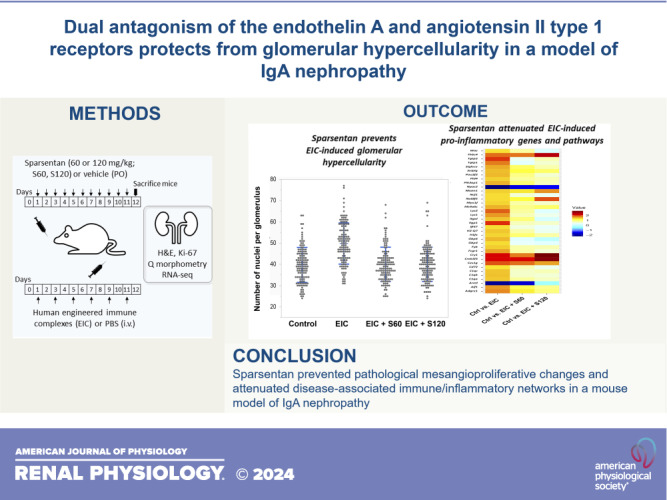

## INTRODUCTION

IgA nephropathy (IgAN) is the most common primary glomerulonephritis worldwide and a significant cause of kidney failure. IgAN, described for the first time in 1968, is diagnosed based on pathological assessment of kidney biopsy tissues with the characteristic glomerular IgA-containing immunodeposits (1, 2). IgA in these immunodeposits is exclusively of the IgA1 subclass (3) and enriched for glycoforms that have some hinge-region *O*-linked glycans without galactose, i.e., galactose-deficient IgA1 (Gd-IgA1) (4–6).

The working hypothesis for pathogenesis of IgAN postulates that Gd-IgA1, often elevated in the circulation of patients with IgAN, is recognized by IgG autoantibodies, resulting in the formation of IgA1-IgG circulating immune complexes. Some of these complexes may escape the usual clearance mechanisms, deposit in the glomeruli, and induce kidney injury (7, 8).

This hypothesis is supported by multiple lines of evidence. For example, serum levels of Gd-IgA1 and the corresponding IgG autoantibodies predict disease progression in patients with IgAN (9, 10). Recently, it was shown that IgG is present in the glomerular immunodeposits of all patients with IgAN. IgG in these immunodeposits, but not in those of patients with other types of glomerular diseases, such as lupus nephritis and membranous nephropathy, is enriched for autoantibodies specific for Gd-IgA1 (11).

The pathogenic potential of immune complexes consisting of Gd-IgA1 and IgG autoantibodies has been confirmed using multiple experimental approaches. For example, IgA1-IgG immune complexes isolated from sera of patients with IgAN activate cultured primary human mesangial cells resulting in their proliferation and overproduction of extracellular matrix proteins and some cytokines/chemokines (12, 13). Furthermore, immune complexes formed in vitro from human Gd-IgA1 and IgG purified from sera of patients with IgAN, when injected into experimental mice, form glomerular immunodeposits and induce mesangioproliferative pathological changes mimicking IgAN. These glomerular deposits consist of human IgA1 and IgG and mouse complement C3 (14). Recently, we have developed a small-animal model using engineered immune complexes (EIC) formed from human Gd-IgA1 and a recombinant human IgG (rIgG) autoantibody specific for Gd-IgA1. In this model, the EIC are formed in vitro and then injected into immunodeficient mice to induce glomerular mesangioproliferative injury (14).

IgAN is a chronic kidney autoimmune disease, with up to 40% of patients developing kidney failure within 20 yr of diagnosis (15, 16). Moreover, IgAN frequently recurs after kidney transplantation (17). Consequently, life expectancy for patients diagnosed with IgAN is reduced (18). Therefore, new therapies are needed to treat patients with this chronic progressive kidney disease.

Beneficial effects of inhibition of endothelin 1 and renin-angiotensin systems in clinical IgAN (19–21) suggest that both pathways are involved in the pathobiology of IgAN, but the combined mechanisms remain unclear. The ongoing phase 3 PROTECT trial demonstrated superior preservation of kidney function and antiproteinuric effects of sparsentan, a novel, orally active, first-in-class single-molecule dual endothelin angiotensin receptor antagonist (DEARA) (22) compared with the angiotensin receptor blocker (ARB) irbesartan in patients with IgAN (21, 23). Sparsentan received accelerated approval in the United States for the reduction of proteinuria in adults with IgAN at high risk of disease progression (23). Although the nephroprotective potential of sparsentan has been demonstrated in the setting of a large clinical trial, the mechanisms of nephroprotection by this drug are still being elucidated.

To understand how sparsentan may affect gene expression in glomerular pathways in IgAN, we used our mouse model of IgAN, wherein administration of EIC induced glomerular mesangioproliferative injury (14) and tested whether sparsentan can prevent or attenuate these pathological changes. We demonstrate that sparsentan provided beneficial effects on glomerular hypercellularity in our mouse model of IgAN and attenuated abnormal expression of genes associated with inflammatory pathways. Similar inflammatory pathways and genes have been shown in multiple independent studies to be enhanced in IgAN kidney-biopsy specimens and also in the early stage of development of the disease in gddY mouse model of IgAN (24–26). Together, these data suggest an important role for the endothelin type A receptor (ET_A_R) and angiotensin II type I receptor (AT_1_R) in mediating the proinflammatory effects of immune complex deposition in the kidneys of patients with IgAN.

## MATERIALS AND METHODS

### Mice

**Figure 1.**
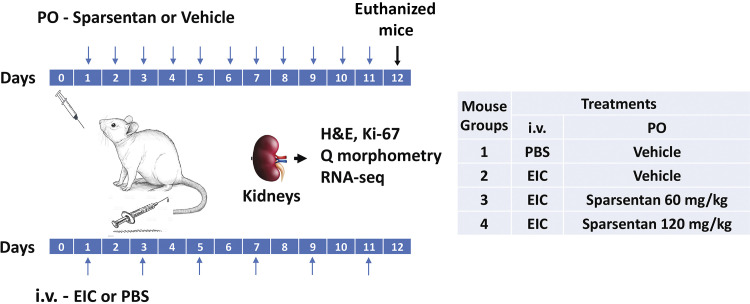
Schematic representation of experimental design. NCr nude mice were injected via the tail vein with human engineered immune complexes (EIC) (*groups 2, 3,* and *4*) or with PBS (*group 1*, Control) every other day from *day 1* to *day 11* (*bottom*: timeline and arrows). Mice also received daily oral gavage (PO) from *day 1* to *day 11* (*top*: timeline with arrows) with either vehicle (*groups 1* and *2*) or sparsentan 60 mg/kg (*group 3*) or sparsentan 120 mg/kg (*group 4*). Mice were euthanized on *day 12*, and kidneys were excised for further analyses. H&E, hematoxylin and eosin stain; iv, intravenous; PBS, phosphate-buffered saline; PO, orally; Q morphometry, quantitative morphometry; RNA-Seq, next-generation sequencing of kidney transcriptome.

NCr nude sp/sp homozygous 7-wk-old female mice were purchased from Taconic Farms (Germantown, NY). Mice were housed in microisolator cages and received sterile food and water at the University of Alabama at Birmingham (UAB; Birmingham, AL) animal facility. After arrival, acclimatization of mice was carried out for a minimum of 3 days. The experimental protocols followed the procedures described in the National Institutes of Health *Guide for Care and Use of Laboratory Animals*, the PHS Policy on Humane Care and Use of Laboratory Animals, the USDA Animal Welfare Act and Regulations, and University policies and Animal Resources Program Standard Operating Procedures. The experimental protocol was approved by the UAB Institutional Animal Care and Use Committee (IACUC). Mice were randomly divided into four groups, of five mice each, with a total of 20 mice. Sample size was based on the prior studies (14). No criteria were set a priori for inclusion or exclusion, and no mice were excluded from analysis. No randomization of mice was performed, and all treatments of mice were done in parallel as detailed in Fig. 1. No control for confounders was used with respect to cages and treatment variations. No adverse events were observed. No end-points were set.

### Experimental Approaches

#### Formation of IgA1-IgG immune complexes in vitro.

Immune complexes were formed from reagents described previously, human Gd-IgA1 and a human recombinant IgG (rIgG) autoantibody specific for Gd-IgA1 (14). We used polymeric IgA1 (Ale) myeloma protein that is naturally galactose-deficient in some *O*-glycans and contains very little sialic acid on these *O*-glycans (27), thus mimicking Gd-IgA1 in IgAN. Importantly, this protein is recognized by IgG autoantibodies extracted from glomerular immune deposits of patients with IgAN (11). Human rIgG autoantibody specific for Gd-IgA1 was produced as previously described (28). The purity and molecular integrity of both proteins were assessed by SDS-PAGE under reducing and nonreducing conditions. EIC were formed in vitro by incubating purified Gd-IgA1 with rIgG autoantibody overnight at 4°C in a proportion of 2:1 (wt:wt) (14). The formation of IgA1-rIgG immune complexes, termed EIC, was confirmed by immune-complex formation assay, as described previously (11).

#### Mouse experiments with EIC and sparsentan.

The in vitro-formed EIC were intravenously injected into the lateral tail vein of NCr nude mice. Each mouse was injected with EIC containing 0.2 mg Gd-IgA1 and 0.1 mg rIgG in approximately 0.1 mL total volume (14). Injections were given every other day for a total of six injections. Negative-control mice did not receive any EIC. In addition, either 60 (group 3; EIC + SP60) or 120 (group 4; EIC + SP120) mg/kg sparsentan in 0.2 mL vehicle (0.25% Tween 80, 0.5% wt/vol methylcellulose in distilled water) were administered daily by oral gavage from the first day of EIC injections for a total of 11 days. Selection of sparsentan doses was made following a pilot study (data not shown). The negative and the positive control mice (groups 1 and 2, respectively) were orally gavaged with 0.2 mL of vehicle alone. On *day 12*, 1 day after the last EIC injection and administration of vehicle or sparsentan, mice were euthanized, and kidneys were excised for histopathology and RNA-Seq analyses. The experimental protocol is illustrated schematically in Fig. 1.

### Histological Examination

One half of a kidney excised from mice on *day 12* (Fig. 1) was fixed in 10% neutral-buffered formalin, processed, and embedded in paraffin. Tissue sections (4-µm thick) were stained with hematoxylin-eosin (H&E) and periodic acid-Schiff (PAS) reagents (14) and examined by a pathologist using light microscopy (Olympus BX60). Images of 25 glomeruli per each mouse kidney stained with H&E were acquired (magnification ×200). Quantitative morphometric analysis was used to evaluate the pathological changes induced by the administration of EIC in mice gavaged with vehicle and compared with control mice (no EIC) and those that received EIC and sparsentan. The number of nuclei per glomerulus was calculated using ImageJ-Fiji software. PAS-stained tissues were examined using light microscopy to assess changes in glomerular extracellular matrix. The pathologist (LN) who performed the quantitative morphometry and histological evaluations was blinded to the identity and treatment group of mice.

Immunohistochemical staining for mesangial proliferation in the glomeruli was performed with a Ki-67 rabbit polyclonal antibody (Abcam, Waltham, MA) followed by anti-rabbit IgG conjugated with horse radish peroxidase (HRP; OneStep Polymer HRP anti-rabbit, GeneTex, Irvine, CA) and visualized with ImmPACT DAB Substrate kit for HRP (Vector Laboratories, Newark, CA). A Ki-67-positive glomerulus was defined as a glomerulus containing at least one Ki-67-stained cell. Up to 210 glomeruli per kidney were assessed, and the number of positive glomeruli was expressed as a percentage of the total number of glomeruli examined.

### Kidney Transcriptomics: Differential Gene Expression and Network Analysis

**Figure 2.**
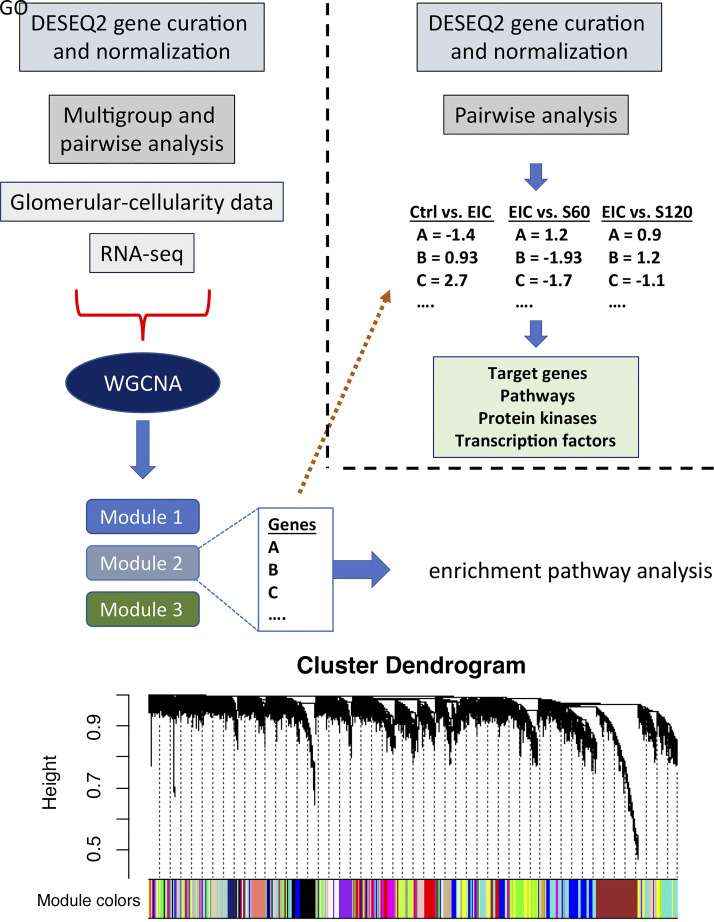
Schematic representation of bioinformatic workflow. NGS mRNA data were normalized and curated for outliers as stated in materials and methods and used for WGCNA. Quantitative morphological data, number of nuclei per glomerulus, and percent of Ki-67-positive glomeruli were incorporated into the WGCNA workflow as the phenotypic comparison data. Module eigengenes were based on gene network clusters and their correlation with the quantitative phenotypic data for each mouse and then within each testing group. Statistically significant modules and their significant genes which correlated to phenotype were then used to assess relevant GO biological process pathways and to determine up- or downregulation of gene sets in those pathways. Finally, upstream protein kinases and downstream transcription factors were imputed from significant gene sets associated with phenotypic outcomes. EIC, engineered human immune complexes; GO, Gene Ontology; NGS, next-generation sequencing; RNA-Seq, next-generation sequencing of kidney transcriptome; S60, sparsentan 60 mg/kg; S120, sparsentan 120 mg/kg; WGCNA, weighted gene correlation network analysis.

We used RNA-Seq to investigate the gene expression changes in the EIC mouse model and to assess the effects of sparsentan in preventing or ameliorating EIC-induced profiles. Analyses were performed both at the single-gene level and at the network level, across the three main group comparisons (Control vs. EIC, EIC vs. EIC + S60, and EIC vs. EIC + S120), as detailed in Fig. 2. We also leveraged the pathological glomerular changes of the EIC-injected animals as quantitative phenotypes of abnormal mesangial proliferation in gene-network correlation analyses to identify the most important networks and hub genes likely involved in such phenotypes. Healthy control and IgAN glomerular biopsy transcriptomes were obtained from public GEO database GSE37463, and their DGE was compared with transcriptional changes in the EIC-injected model (29).

Kidney tissues from experimental animals stored in RNAlater at −80°C were sent to GeneWiz (South Plainfield NJ) for RNA-Seq analysis (RIN and alignment details in the Supplemental Material). Workflow for bioinformatic analysis is illustrated schematically in Fig. 2. RNA-Seq raw data were processed using the DESeq2 R package to normalize, log_2_ scale count data, and filter out low-expressing genes (reads <10). About 15,000 unique genes were analyzed for network-level profiling to identify groups of coexpressed genes (modules) associated with glomerular proliferation (Q-morphometry; cell nuclei per glomerulus). We used the weighted gene correlation network analysis (WGCNA) (30) R package with unsigned networks. Functional networks are defined by the clustering of genes within WGCNA modules based on their coexpression pattern, and herein are called gene modules or networks interchangeably. A power level of 7 was used to transform gene Pearson correlation coefficients to generate scale-free topology using the soft-thresholding function. The minimum number of genes in a module was kept to the default value of 30 (default parameters for unsigned analysis). The gene expression variance of each module was summarized with the eigengene (ME) function, thus reducing the expression profile of each module to a single expression value that can be associated with phenotype/traits. For those gene modules that significantly associated with the cellular phenotypes, we also calculated two gene expression features that are typically used to identify hub genes in coexpression network analyses [gene significance (GS) and module membership (MM)]. Only genes with statistically significant GS and MM (*P* < 0.05 for both) were identified as critical hub genes across MEs that were correlated to the phenotype of interest. Topologically, hub genes tend to be positioned at the center of a functional network and are considered the main drivers of associations with phenotypes. Using MEs that significantly correlated with glomerular proliferation (*P* < 0.05), we grouped all statistically significant genes from those modules (GS, *P* < 0.05) and cross-referenced their respective single-gene level changes calculated from the differential gene expression (DGE) analysis performed with DESeq2. Thus, we used their respective log fold change and *P*-adj values. Volcano plots of DE genes are shown in Supplemental Fig. S1. Expression level changes were graphically represented in heatmaps (31).

Gene set enrichment analysis (GSEA) Gene Ontology (GO) biological processes were used to assess significant differential changes at the pathway level in three different pairwise-group analyses (Control vs. EIC, EIC vs. EIC-S60, and EIC vs. EIC-S120) (gsea-msigdb.org) (32, 33). Genes with statistical significance identified in trait-correlated MEs by WGCNA (outlined above) were grouped and uploaded for analysis. The top 10 pathways were selected for presentation. The direction of the pathways was determined by cross referencing the genes identified by WGCNA with the log_2_ fold change (LFC) determined by DESeq2 LFC. Pathway enrichment *P* values were adjusted with FDR. Using top LFC hub genes identified from the WGCNA analysis as input for the Xpression2Kinases (X2K) software, we imputed a correlation of upstream cell signaling kinase networks with downstream transcription factors (34, 35). This computational tool (Ma’ayan Laboratory, Icahn School of Medicine at Mount Sinai, New York, NY) uses chromatin immunoprecipitation data, protein-protein interactions, and known kinase-substrate interactions to impute network connections based on differential gene expression data.

### Quantitative RT-PCR

RNA was extracted from the mouse kidney tissues (see Fig. 1) and used to synthesize cDNA for qPCR experiments by using Fast SYBR Green mix (Thermo Fisher, Scientific, Waltham, MA) with the gene-specific primers and Bio-Rad C-1000 Thermal cycler (Hercules, CA) as detailed in the Supplemental Material.

### Statistical Analysis

Statistical significance of data from quantitative morphometric analysis between groups was analyzed by ANOVA. Data are expressed as mean or median ± SD values. *P* < 0.05 was considered statistically significant. All statistical analyses were performed using JMP Pro (Cary, NC). To test for probability of false positives between module eigengenes and glomerular proliferation, we ran 200 permutations scrambling the gene module data, and compared the *Z*-scores to the real data. We found for the Control (PBS) versus EIC a probability of 0.015 and for EIC versus EIC-60 and EIC versus EIC120 a probability of *P* < 0.01 for a type 1 error.

qPCR raw counts were normalized using the average value across biological replicates of the reference gene *Gapdh*. Fold changes (FC) were calculated as the ratio of the normalized gene expression (2^−ΔCt^) in the Test Sample to the normalized gene expression (2^−ΔCt^) in the Control Sample. *P* values were calculated based on a one-way ANOVA of the replicate 2^−ΔCt^ values for each gene in the control group and test groups, and unadjusted *P* values < 0.05 were considered significant.

## RESULTS

### Sparsentan Protected Against Glomerular Pathological Changes Induced by IgA1-IgG Immune Complexes

**Figure 3.**
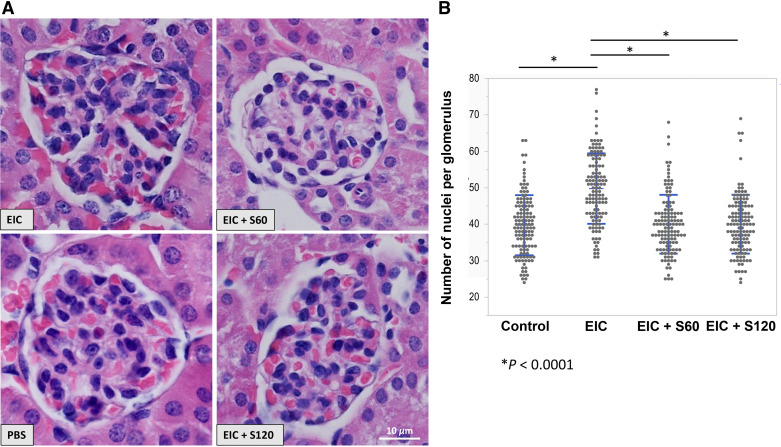
Quantitative morphometric evaluation of pathological changes in glomeruli. Kidney tissue obtained from the mice treated as described in Fig. 1. microscopically analyzed for pathological changes. *A*: examples of hematoxylin and eosin-stained kidney-tissue sections are shown. Images were taken with ×60 objective, size bar marks 10 µm (*bottom right*). *Top left*, *group 2* (EIC-injected mice; 54 nuclei per glomerulus); *bottom left*, *group 1* (PBS, i.e., control; 42 nuclei per glomerulus); *top right*, *group 3* [EIC + S60 (sparsentan 60 mg/kg); 40 nuclei per glomerulus]; and *bottom right*, *group 4* [EIC + S120 (sparsentan 120 mg/kg); 44 nuclei per glomerulus]. *B*: number of nuclei per glomerulus for the four groups of mice. Individual values and means ± SD are shown for each group. *P < 0.0001 for EIC vs. Control or EIC+S60 or EIC+S120 for each pairwise comparison. Conversely, comparisons for Control vs. EIC+S60 or EIC+S120 did not show statistically significant differences (ANOVA, *n* = 5). EIC, engineered human immune complexes; PBS, phosphate-buffered saline; S60, sparsentan 60 mg/kg; S120, sparsentan 120 mg/kg; SD, standard deviation.

**Figure 4.**
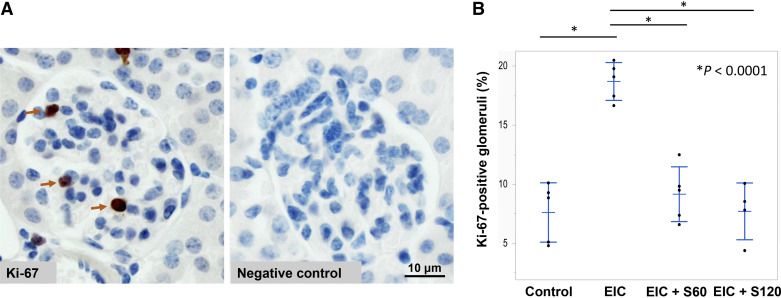
Sparsentan prevents EIC-induced glomerular cellular proliferation. *A*: representative examples of Ki-67 immunohistochemical staining. The number of glomeruli containing actively proliferating cells was determined by immunostaining using a rabbit antibody specific for Ki-67 followed by a secondary anti-rabbit antibody, as detailed in materials and methods. *Left* shows a glomerulus from a mouse injected with EIC that has three Ki-67-positive cells (marked by arrows), *right* is a negative control (no Ki-67-specific antibody, only the secondary antibody) confirming specificity of the staining. *B*: percent of Ki-67-positive glomeruli in the four groups of mice. Data are shown for individual mice as mean values, bars show means ± SD values for each group. A Ki-67-positive glomerulus was defined as any glomerulus that had at least one Ki-67-positive cell. Up to 210 glomeruli were assessed per mouse and the number of Ki-67-positive glomeruli expressed as a percent of the total number of glomeruli examined. *P < 0.0001 for EIC vs. Control or EIC+S60 or EIC+S120 for each pairwise comparison. Conversely, comparisons for Control vs. EIC+S60 or EIC+S120 were not statistically significant (ANOVA, *n* = 5). Note: one of five mice in EIC+S120 group did not exhibit any Ki-67 staining in any glomeruli and was excluded from the analyses. EIC, engineered human immune complexes; S60, sparsentan 60 mg/kg; S120, sparsentan 120 mg/kg.

EIC injections increased glomerular cellularity compared with the control mice that received only PBS (*P* < 0.0001) (Fig. 3, *A* and *B*). Sparsentan at 60 or 120 mg/kg prevented these EIC-induced pathological changes (*P* < 0.0001 when compared with EIC-injected mice) (Fig. 3, *A* and *B*). Sparsentan also prevented the EIC-induced increase in actively proliferating mesangial cells, as evidenced by Ki-67 staining of mouse kidney tissues (Fig. 4, *A* and *B*). Evaluation of pairwise comparison data showed statistically significant differences (*P* < 0.0001) for EIC-treated mice compared with control, EIC + S60, or EIC + S120. Conversely, Ki-67-positive glomeruli in control mice compared with EIC + S60 or EIC + S120 were not statistically significantly different (Fig. 4, *A* and *B*), indicating that sparsentan treatment prevented EIC-induced glomerular proliferation.

### Sparsentan Treatment Ameliorated the Activated Proinflammatory Genes and Pathways Induced in Mouse Kidneys by EIC

**Figure 5.**
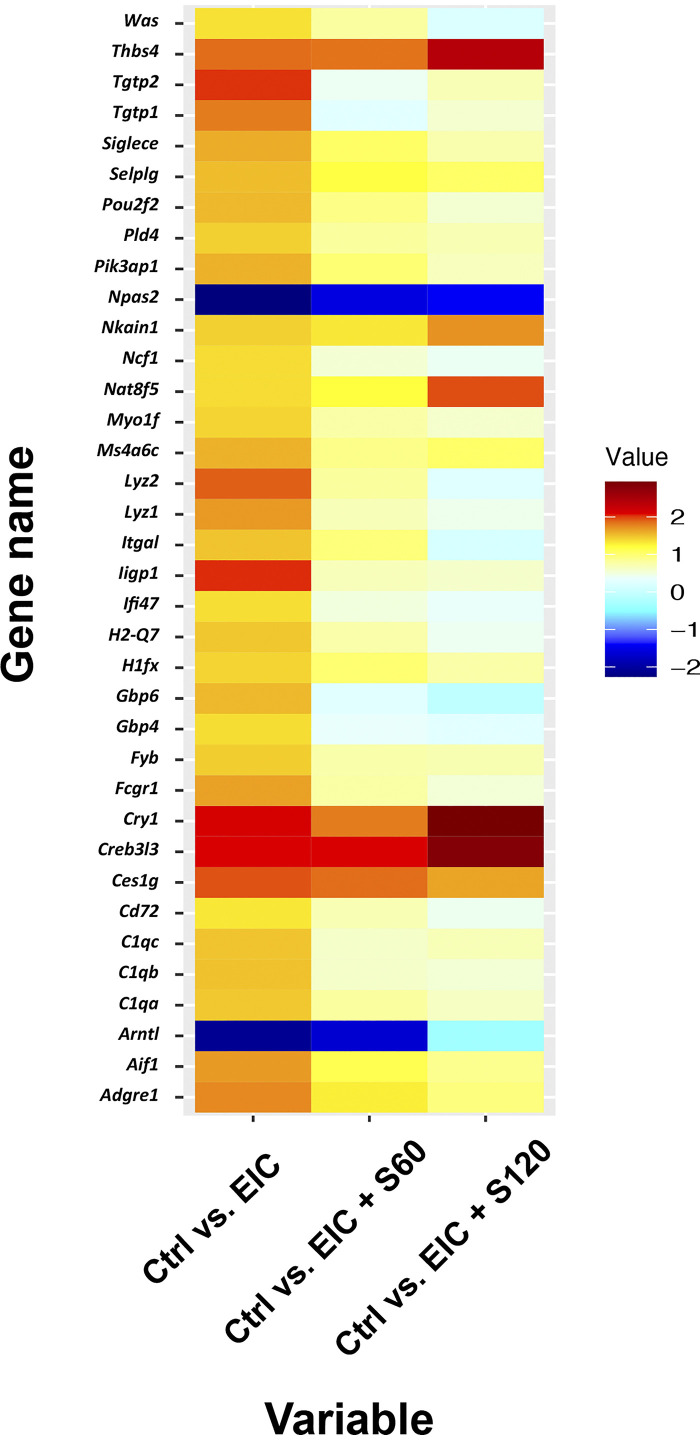
Top WGCNA module genes show normalization or reversal of expression after sparsentan treatment. WGCNA between grouped pairs (Control vs. EIC, Control vs. EIC+S60, Control vs. EIC+S120) was performed to find module eigengenes that correlate with glomerular phenotypes (nuclei per glomerulus and Ki-67 positivity). Genes from modules significantly associated with glomerular phenotypes (*P-*adj < 0.05) were then cross-referenced and compared with differential expression analysis for sequence 2 (DESeq2) log_2_ fold change analysis. A heatmap was generated using these statistically significant genes for the ME-nuclei/glomerulus phenotype. Ctrl, control; EIC, engineered immune complexes; ME, module eigengene; S60, sparsentan 60 mg/kg; S120, sparsentan 120 mg/kg; WGCNA, weighted gene correlation network analysis.

We performed network-level analysis (WGCNA) to identify changes in disease-relevant pathways associated with the EIC-induced cellular phenotypes across the main group comparisons (Control vs. EIC, EIC vs. EIC + S60, and EIC vs. EIC + S120), as well as Control versus EIC + S60 or EIC + S120. We identified 33 gene modules that significantly associated (*P* < 0.05) with glomerular proliferation (number of nuclei per glomerulus) and Ki-67-positive glomeruli (Supplemental Fig. S2) and demonstrated that associations were not due to chance alone (Supplemental Fig. S3). As network perturbations can largely be attributed to alteration in expression levels of a few centrally located and functionally related genes or hubs, we sought to pinpoint which hub gene showed the greatest level of dysregulation within each module associated with the cellular phenotypes. DGE analysis of top modules hub genes in the kidneys of EIC-injected mice compared with control mice (Control) showed that the greatest gene-level dysregulation was in genes with immune and cytokine signaling functions. These genes were found to be upregulated, with the top LFCs ranging from 1.66 to 2.78 (Table 1) and included the T cell-specific GTPases *Tgtp1* (1.79 LFC) and *Tgtp2* (2.02 LFC). We performed the same DGE analysis with top network hub genes with the two sparsentan-treated, EIC-injected mouse groups (EIC + S60 and EIC + S120) compared with the untreated EIC group. We found that sparsentan at 60 or 120 mg/kg led to an attenuation of expression levels of immune-related genes including *Tgtp1* (−1.51 LFC for EIC vs. EIC + S60) and *Tgtp2* (−1.63 and −1.35 LFC for EIC vs. EIC +S60 or S120, respectively) (Table 1, Fig. 5).

**Table 1.** Top LFC in gene expression in DESeq2 identified from top modules in WGCNA

Control vs. EIC		EIC vs. EIC + S60		EIC vs. EIC + S120	
Gene	LFC	Gene	LFC	Gene	LFC
Z-DNA binding protein 1 (*Zbp1*)	2.78	***Tgtp2 ***	−1.63	***Fcgr4 ***	−1.90
C-type lectin domain family 4, member a1 (*Clec4a1*)	2.46	***Gm4951 ***	−1.62	***Myo1g ***	−1.70
Fc receptor IgG IV (*Fcgr4*)	2.05	***Tgtp1 ***	−1.51	***Nlrc5 ***	−1.68
Interferon-inducible GTPase 1 (*Iigp1*)	2.04	***Iigp1 ***	−1.39	***Lyz2*** * *	−1.65
T cell-specific guanine nucleotide triphosphate-binding protein 2 (*Tgtp2*)	2.02	***Fcgr4 ***	−1.35	*Gbp6 *	−1.61
Lysozyme 2 (*Lyz2*)	1.90	***Nlrc5 ***	−1.34	***Clec4a1 ***	−1.51
Myosin 1 G (*Myo1G*)	1.86	Guanylate binding protein family member 6 (*Gbp6*)	−1.26	***Iigp1 ***	−1.46
NOD-like receptor family CARD domain containing 5 (*Nlrc5*)	1.81	C-type lectin domain family 12 member A (*Cleck12a*)	−1.23	Neutrophil cytosolic factor 4 (*Ncf4*)	−1.43
T cell-specific guanine nucleotide triphosphate-binding protein 1 (*Tgtp1*)	1.79	***Zbp1 ***	−1.12	Mitogen-activated protein kinase kinase kinase kinase 1 (*Map4k1*)	−1.36
Interferon-gamma-inducible GTPase Ifgga2 protein (*Gm4951*)	1.79	***Ms4a6b*** * *	−1.11	***Tgtp2 ***	−1.35
Adhesion G protein-coupled receptor E1 (*Adgre1*)	1.74	***Lyz2 ***	−1.10	Integrin subunit alpha L (*Itgal*)	−1.33
C-type lectin domain family 4, member a2 (*Clec4a2*)	1.74	Guanylate-binding protein 9 (*Gbp9*)	−1.09	***Clec4a2 ***	−1.27
Membrane-spanning 4-domains subfamily A member 6B (*Ms4a6b*)	1.70	Tryptase beta 2 (*Tpsb2*)	−1.04	***Lyz1 ***	−1.25
Lysozyme C-1 (*Lyz1*)	1.67	XIAP-associated factor 1 (*Xaf1*)	−1.03	Integrin beta 2 *(Itgb2*)* *	−1.24
Allograft Inflammatory Factor 1 (*Aif1*)	1.66	Schlafen family member 8 (*Slfn8*)	−1.03	*Xaf1 *	−1.24

WGCNA between grouped pairs (Control vs. EIC, EIC vs. EIC + S120, EIC vs. EIC + S60) was performed to find module eigengenes that correlate with nuclei per glomerulus and Ki-67 positivity. Combined genes from WGCNA top modules were cross-referenced with DESeq2 data from those groups, and genes with highest LFC changes are shown. Comparison of the top genes; LFC in bold indicates a top gene also found in control vs. EIC group. DESeq2, differential expression analysis for sequence 2; EIC, engineered immune complexes; LFC, log_2_ fold change; S60, sparsentan 60 mg/kg; S120, sparsentan 120 mg/kg; WGCNA, weighted gene correlation network analysis.

The cross-referencing of DE genes and gene modules from the network analysis indicated that sparsentan treatment largely prevented dysregulation in the top 36 genes, ranked based on highest module correlation. The comparisons performed between Control versus EIC, Control versus EIC + S60, and Control versus EIC + S120 demonstrated that sparsentan treatment promoted reversal of EIC-induced expression levels toward those of the control mice. For example, increased expression of the C1q components of the classical complement pathway (*C1qa, C1qb, C1qc*) observed in the EIC-alone group was attenuated by sparsentan treatment. Moreover, in cases where genes were repressed, such as the mammalian clock gene regulatory network (*Arntl*), EIC-induced expression changes were also prevented (Fig. 5).

**Figure 6.**
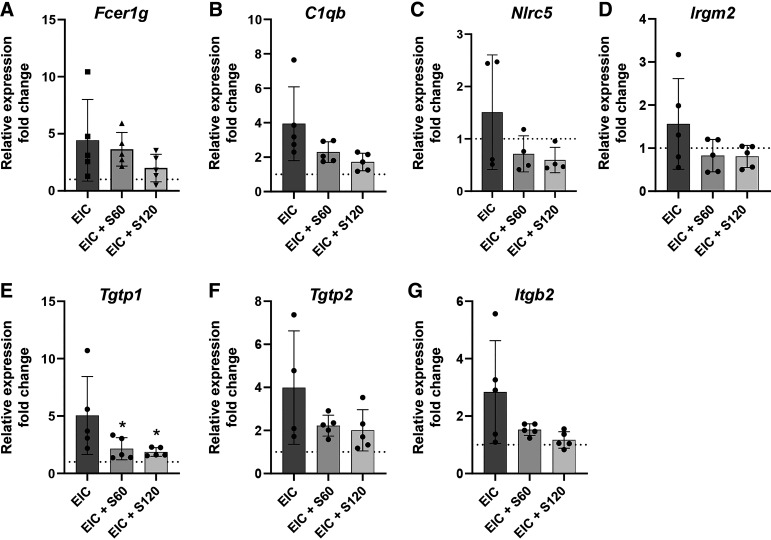
Validation of gene expression changes by qRT-PCR. To validate gene-expression changes determined by NGS (RNA-Seq), gene expression of a subset of hub genes was assessed using qRT-PCR. We used the following target genes: *Fcer1g* (Fc epsilon receptor Ig; *A*), *C1qb* (complement C1q B chain; *B*), *Nlrc5* (NLR family CARD domain containing 5; *C*), *Irgm2* (immunity-related GTPase family M member 2; *D*), *Tgtp1* (T cell-specific GTPase 1; *E*), *Tgtp2* (T cell-specific GTPase 2; *F*), and *Itgb2* (integrin subunit beta 2; *G*). Genes encoding *β-actin and GAPDH* (glyceraldehyde-3-phosphate dehydrogenase) were used for normalization. All gene expression data are shown as ΔΔCt, calculated as log_2_ fold change vs. expression levels of that gene in control mice. Dashed line at 1 indicates where no change is found from control expression level. *P < 0.05 vs. EIC, *n* = 5 for each sample set. EIC, engineered immune complexes; NGS, next-generation sequencing; qRT-PCR, quantitative reverse transcription polymerase chain reaction; S60, sparsentan 60 mg/kg; S120, sparsentan 120 mg/kg.

qRT-PCR targeting a subset of hub genes, *Fcer1g*, *C1qb*, *Nlrc5*, *Irgm2*, *Tgtp1*, *Tgtp2*, and *Itgb2*, showed a consistent increase of expression in mice injected with EIC compared with the control mice, thus validating the gene expression alterations identified in our network-level analysis from the NGS data. The EIC-induced upregulation of the genes examined was ameliorated by sparsentan at both doses, with a clear trend for a lessening of expression following sparsentan treatment compared with that in control mice (Fig. 6). Notably the expression of *Tgtp1* was statistically significantly lower (*P* < 0.05) in kidneys from EIC + SP60 and EIC + SP120 compared with the EIC group (Fig. 6).

The effect of EIC and sparsentan on disease-relevant pathways using GSEA revealed that immune- and inflammatory-related pathways, such as “cytokine-mediated signaling,” “cellular response to type 1 interferon,” and “type I interferon signaling” were the most significant pathways when all three pairwise datasets were analyzed. EIC increased expression of genes in these pathways and sparsentan treatment attenuated the pathway-specific gene expression (Tables 1 and 2).

**Table 2.** Pathway-level analysis shows downregulated expression of genes involved in immune/inflammatory processes after sparsentan treatment

Up Pathways		Down Pathways			
Control vs. EIC		EIC vs. EIC+S120		EIC vs. EIC+S60	
Pathway	*P* Value	Pathway	*P* Value	Pathway	*P* Value
Cytokine-mediated signaling pathway	2.53E-08	Cytokine-mediated signaling pathway	2.97E-09	Cellular response to type I interferon	1.49E-08
Cellular response to type I interferon	2.53E-08	Cellular response to type I interferon	3.87E-09	Type I interferon signaling pathway	1.49E-08
Type I interferon signaling pathway	2.53E-08	Type I interferon signaling pathway	3.87E-09	Cytokine-mediated signaling pathway	2.90E-07
Neutrophil activation involved in immune response	3.52E-05	Neutrophil activation involved in immune response	1.24E-05	Neutrophil activation involved in immune response	0.001
Neutrophil degranulation	6.37E-05	Neutrophil degranulation	2.40E-05	Neutrophil degranulation	0.002
Neutrophil-mediated immunity	8.22E-05	Neutrophil-mediated immunity (GO:0002446)	3.08E-05	Antigen processing and presentation of exogenous peptide antigen via MHC class I	0.002
Cellular response to cytokine stimulus	7.33E-04	Cellular response to cytokine stimulus	0.001	Neutrophil-mediated immunity	0.002
Antigen processing and presentation of exogenous peptide antigen via MHC class I	0.003	Antigen processing and presentation of exogenous peptide antigen via MHC class I	0.001	Response to interferon-gamma	0.003
Response to interferon-gamma	0.004	Response to interferon-gamma	0.001	Response to interferon-beta	0.005
Response to cytokine	0.004	Antigen processing and presentation of exogenous peptide antigen via MHC class I, tap-dependent	0.003	Antigen processing and presentation of exogenous peptide antigen via MHC class I, tap-dependent)	0.005

WGCNA analysis between grouped pairs (Control vs. EIC, EIC vs. EIC + S120, EIC vs. EIC + S60) was performed to find module eigengenes that correlate with glomerular phenotypes (nuclei per glomerulus and Ki-67 positivity). Genes from the modules significantly associated with the cellular phenotypes were then grouped and subjected to GSEA GO biological process pathway analysis. Top 10 pathways for each pairwise analysis are shown. LFC of genes within each pathway were identified using DESeq2 data and found to be decreased compared with the LFC of the upregulated genes in control vs. EIC. DESeq2, differential expression analysis for sequence 2; EIC, engineered immune complexes; GO, gene ontology; GSEA, gene set enrichment analysis; INF, interferon; LFC, log_2_ fold change; MHC1, major histocompatibility complex class I; S60, sparsentan 60 mg/kg; S120, sparsentan 120 mg/kg; WGCNA, weighted gene correlation network analysis.

### Upstream Regulatory Network Imputation Revealed Changes in Protein Kinases and Transcription Factors in Kidney Tissues of Mice Injected With EIC Compared With Mice That Received EIC and Sparsentan

**Figure 7.**
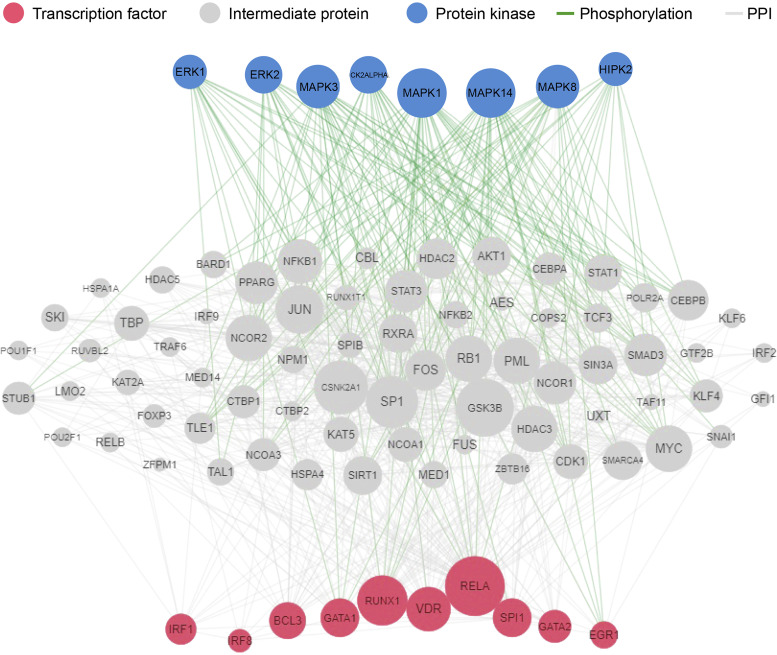
WGCNA top module genes: protein-kinase and transcription-factor analysis for EIC-injected mice vs. control mice. Significantly correlated genes compared with the ME identified from top ME-trait correlations in the EIC vs. control group were uploaded to X2Kweb, a web tool developed for upstream regulatory network inference (maayanlab.cloud/X2K/). This network depicts inferred protein kinases (blue circles), transcription factors (red circles), and intermediate proteins (gray circles) that are known to be involved in regulation of gene lists of interest. PPI is shown with the gray line, and phosphorylation is shown with a green line. EIC, engineered immune complexes; ME, module eigengene; PPI, protein-protein interaction; WGCNA, weighted gene coexpression network analysis.

To gain a deeper understanding on how the identified hub genes are potentially regulated upstream by well-known protein kinases and transcription factors, we leveraged the X2K database. In the EIC-injected mice without sparsentan administration, we found that MAPK1/3/8/14 were the top associated protein kinases, with the top transcription factors *RelA* (NF-κB cofactor), vitamin D receptor (*Vdr*), and runt-related transcription factor 1 (*Runx1*) (Fig. 7). In EIC + S60 versus control group, the top protein kinase associations included GSK3B, CDK1, and MAPK14, and the top transcription factors myelocytomatosis oncogene (*Myc*), peroxisome proliferator-activated receptor gamma (*Pparg*), and Krüppel-like factor 4 (*Klf4*) (Supplemental Fig. S4*A*). In EIC + S120 versus control, the top protein kinase associations included CDK1, CSNK2A1, and MAPK14, and the top transcription factors were E2 alpha (*Tcf3*), Sin3a, and sex-determining region Y box 2 (*Sox2*) (Supplemental Fig. S4*B*). Animals treated with sparsentan showed a shift in the signaling pathways regulating the downstream hub genes that control the mesangial proliferation. These include some of the MAPKs, which are critical master regulators for controlling cell proliferation during states of stimulation or damage (36).

### Many EIC-Dysregulated and Sparsentan-Responsive Genes With Immune/Inflammatory Functions Are Dysregulated in Kidney Tissues of Patients With IgAN

**Figure 8.**
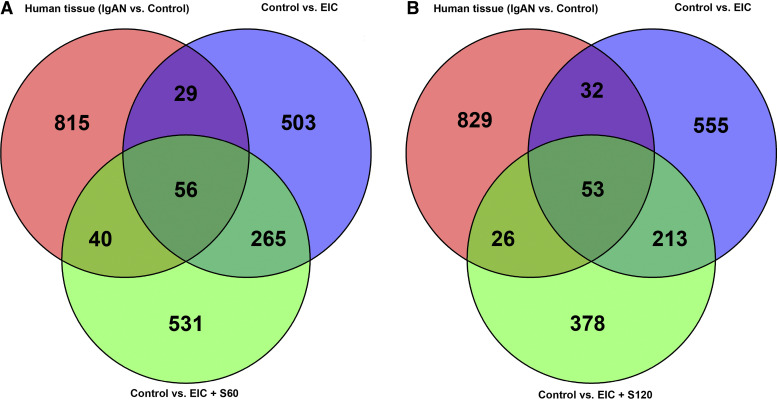
Gene networks in IgAN human-kidney tissue partially overlap with dysregulation in an EIC animal model treated with sparsentan. Transcriptional data of microdissected glomeruli from renal-biopsy specimens of patients with IgAN and controls were analyzed using differential expression analysis for sequence 2 (DESeq2).(25) Statistically significant genes were compared in a Venn diagram to top weighted gene correlation network analysis (WGCNA) module eigengene genes with *P-*adj < 0.05 correlation from multiple pairwise analyses. *A*: human tissue, Control vs. EIC, Control vs. EIC+S60, human tissue (*B*), Control vs. EIC, Control vs. EIC+S120. DESeq2, differential expression analysis for sequence 2; EIC, engineered immune complexes; IgAN, immunoglobulin A nephropathy; S60, sparsentan 60 mg/kg; S120, sparsentan 120 mg/kg.

We sought to identify which genes and pathway from our EIC-based animal model may mirror gene expression changes identified in patients with IgAN, thus providing some insights into which of the expression profiles we identified are the most translatable. We performed these analyses based on previously published statistically significant DE genes in microdissected glomeruli from kidney biopsy specimens of patients with IgAN and healthy controls (pretransplant kidney biopsy) (29). The results showed an overlap of 85 DE genes between the glomeruli from patients with IgAN and in mice treated with EIC compared with the respective experimental control baseline. Moreover, in mice treated with EIC + S60, or EIC + S120, 56 and 53 DE genes, respectively, of those 85 genes overlap with the IgAN glomeruli genes and the EIC genes (Fig. 8, *A* and *B*). The overlapping genes between both sparsentan-treated mouse groups were largely similar and included mostly genes involved in immune functions and inflammation including components of the complement pathway (Supplemental Table S1). Notably, three hub genes (*Fcer1g*, *C1qb*, and *Itgb2*) that we identified to be overexpressed in the EIC model and validated to respond to sparsentan were also DE in patients with IgAN, thus further supporting the translatability of our findings. GO Biological Pathway analysis of the overlapping genes showed that the top five pathways for the lower dose of sparsentan (S60) included “immune response,” “regulation of immune responses,” “defense response,” “regulation of immune system process,” and “cell activation” (Supplemental Tables S2 and S3).

## DISCUSSION

In this study, we report that in a mouse model of IgAN, in which engineered human immune complexes are injected to induce mesangioproliferative changes, the treatment with sparsentan prevents this kidney pathology. Although inhibition of RAS with ACE inhibitors or ARBs is considered to be current standard of care in IgAN, simultaneous inhibition of ET-1 may provide additional benefit as recently demonstrated by analysis of PROTECT trial, which compared sparsentan with the ARB irbesartan (21, 23). Targeting the ET_A_Rs alone has been shown to be beneficial in patients with CKD and experimental models of kidney diseases including IgAN (19, 37, 38). The mRNA expression levels of both ET-1 and the renin-angiotensin pathway have been reported to be increased in kidney-biopsy tissue of patients with IgAN (39, 40), and the risk of progression was found to be associated with higher intrarenal expression of ET-1 and angiotensin II.

Gene expression and network analysis of mouse kidney tissues indicated that the mechanisms by which sparsentan exerts its efficacy may be, in part, a consequence of preventing the activation of immune and inflammatory pathways. Notably, these pathways in the animal model were found to be similar to those derived from the genes overlapping with those dysregulated in glomeruli of patients with IgAN compared with the animal models (see Supplemental Fig. S4, *A* and *B*). Although in our mouse model, administration of sparsentan was concomitant with immune complex injections, and differed from how treatment may be initiated relative to disease onset in a clinical course setting, the presence of inflammatory pathways in both the mouse model and biopsy tissue indicates the importance of these mechanisms across the progression of disease pathobiology.

Development of IgAN is thought to result from a combination of several events, or “hits” (41), in genetically susceptible individuals. The first two hits are elevated circulating levels of Gd-IgA1 and production of autoantibodies specific for these IgA1 glycoforms. The third and fourth hits include formation of the IgA1-containing immune complexes and their deposition in the glomeruli with the subsequent activation of mesangial cells, leading to kidney injury. Multiple pathways are likely associated with this immune complex-induced cellular proliferation of mesangial cells. These pathways may involve endothelin 1 and the renin-angiotensin system (42). Analysis of transcriptional signatures in microdissected glomeruli from patients with IgAN revealed association between elevated expression of endothelin 1 (*EDN1*) and disease progression (39). Endothelin 1 and the ET_A_R have been implicated in multiple chronic kidney diseases, and receptor activation is strongly associated with activation of the canonical NF-κB inflammatory pathway (37, 43–45).

In this study, sparsentan treatment of mice injected with EIC not only attenuated the mesangial cell proliferation (Figs. 3 and 4) but also prevented changes observed in transcriptional activities of genes and networks induced by the immune complexes in mouse kidneys. Although *Edn1* expression was not elevated in EIC-injected mice, WGCNA and DGE transcriptional analyses revealed increased expression of proinflammatory genes and pathways in EIC-injected animals that were prevented by sparsentan (Table 1, Supplemental Tables S1– S3). Similar inflammatory-associated pathways were also upregulated in another model of early IgAN, gddY mouse model, using single-cell analytics (26).

The role of complement in IgAN is becoming well recognized, and the amount of complement C3 and detection of mannan-binding lectin in the glomerular immuno-deposits of patients with IgAN are associated with worse renal outcomes (46–51). Although these studies indicate that the complement deposition renally is likely extrarenal in origin, there is also mounting evidence of expression and production of complement components in IgA1 immune complex-activated mesangial cells (52, 53). We found significant upregulation of complement genes, such as *C1qa*, *C1qb*, and *C1qc* in the EIC-injected mouse group, which was prevented by sparsentan treatment (Fig. 5). Two of these genes (*C1qa* and *C1qb*) overlapped in our comparison of DGE profiles of microdissected glomeruli from patients with IgAN, further highlighting the potential role of complement in IgAN pathobiology (Supplemental Table S1) (29). Further support came from proteomic analysis of microdissected glomeruli from human kidney-biopsy specimens from patients with IgAN. These analyses revealed multiple complement proteins, including complement components C1qA, C1qB, and C1qC (54).

Further analysis allowed us to infer that the predominant kinases involved in transcriptional network activity after EIC injection were the associated MAPK classes. Specifically, MAPK1, MAPK14, MAPK3, and MAPK8 were all top targets, and reflected prior findings from IgAN-kidney tissues and in vitro studies (Fig. 7) (55, 56). After sparsentan treatment, the top three kinases identified for EIC + S60 were GSK3B, CDK1, MAPK14, and for EIC + S120 it was CSNK2A1, CDK1, and MAPK14, suggesting that antagonism of the AT_1_R and ET_A_R may potentially modulate the MAPK signaling pathways downstream of EIC (Supplemental Fig. S4, *A* and *B*).

In cultured human mesangial cells, IgA1-containing immune complexes upregulated expression and secretion of proinflammatory cytokines, complement components, and growth factors, such as IL-6, IFN-γ, C3, and platelet-derived growth factor (PDGF) (57, 58). In addition, single-cell transcriptome analysis using kidney biopsy tissues from patients with IgAN and healthy controls revealed an upregulation of multiple inflammatory and ECM-related genes in mesangial cells from patients with IgAN (25). In line with these findings, in our mouse model of IgAN, we identified enhanced expression of inflammation-related genes and pathways in kidney tissues, which was prevented by sparsentan treatment (Table 2).

Comparison of our mouse kidney tissue data with transcriptomic profiling of kidney biopsy specimens from patients with IgAN and controls previously deposited in the GEO database (29) revealed a substantial overlap between DGE expression direction and WGCNA hub genes. In addition, a recent transcriptomic study from renal glomerular biopsy specimens of patients with IgAN reported three hub genes (*ITGB2*, *FCER1G*, and *CSF1R*), two of which were relevant in our model (59). Our data revealed that *Itgb2* and *Fcer1g* were strong hubs (Supplemental Table S4), were significantly upregulated in EIC-injected mice compared with control mice, and were also identified in our GEO-derived biopsy dataset. Notably, their dysregulated expression was normalized in mice receiving sparsentan treatment. Within our model-to-biopsy comparison, most genes that overlapped in this analysis were directionally the same in IgAN and EIC-injected mice, whereas sparsentan attenuated these gene expression changes (Supplemental Table S1). Two of these genes were encoded in the loci identified in a recent GWAS of IgAN, *ITGAM/ITGAX,* and *LY86* loci (60). Pathway analysis of these overlapping genes found that they were all involved in immune-response functions (Supplemental Table S2 and S3). *ITGAM/ITGAX* is expressed by monocytes and other cells and the IgAN-risk alleles were associated with upregulation of *ITGAX* (60). *LY86* gene product, together with CD180 and TLR4, is expressed in monocytes and B cells, mediates the innate immune response to bacterial lipopolysaccharides, and has pleiotropic effects with other GWAS loci on cellular proliferation and immunoglobulin production (60).

Although the analysis of bulk tissue may present with limitations to the identification of a cell type or cell types that carry most of the transcriptional dysregulation, we leveraged a powerful bioinformatics strategy to narrow down the search space of the possible molecular processes underlying the mesangial hyperproliferative phenotype. It is possible that follow-up studies will be able to deconvolute either bioinformatically or with single-cell transcriptomic and refine or add valuable insights to our findings.

Data in the present study suggest that renal protective actions of sparsentan in this disease may involve effects on mesangial cell proliferation in association with complex anti-inflammatory actions. Key inflammatory and proliferative biological elements and pathways that are upregulated in this EIC model of IgAN are markedly normalized by sparsentan. Notable examples include complement genes, integrin components, and Fc receptor elements which may be responsive to activation changes in members of the MAP kinase family. Overlap between mouse and human DEG further supports the hypothesis that sparsentan targets multiple pathological cellular processes in IgAN including the immune and inflammatory processes induced by IgA-IgG immune complexes in the kidneys. These effects of sparsentan are likely the basis of the beneficial effects of the drug in patients with IgAN.

## DATA AVAILABILITY

Raw and curated data are available at the Geo Expression Omnibus under Accession No. GSE225447. Other data and information are available in the Supplemental Material.

## SUPPLEMENTAL DATA

10.6084/m9.figshare.25152839.v2 Supplemental Figs. S1–S4 and Supplemental Tables S1–S4: https://doi.org/10.6084/m9.figshare.25152839.v2

## GRANTS

This work was supported by Travere Therapeutics, Inc.

## DISCLAIMERS

Travere Therapeutics, Inc., provided funding for the study and contributed to discussions on study design, analysis of RNA-Seq data, and editing and review of the manuscript.

## DISCLOSURES

Z.M. and J.N. are coinventors on US patent application 14/318,082 [assigned to the UAB Research Foundation (UABRF)] and licensed by UABRF to Reliant Glycosciences, LLC. J.N. is a co-founder and co-owner of and consultant for Reliant Glycosciences, LLC. C.P.J., R.K., and T.P. are full-time employees of Travere Therapeutics, Inc., and may have an equity or other financial interest in Travere Therapeutics, Inc. Support for efforts and experimental supplies was provided by Travere Therapeutics, Inc. None of the other authors has any conflicts of interest, financial or otherwise, to disclose.

## AUTHOR CONTRIBUTIONS

C.R., Z.M., T.P., S.H., Z-Q.H., T.R., R.K., C.P.J., and J.N. conceived and designed research; C.R., Z.M., T.P., S.H., Z-Q.H., T.R., L.N., and R.K. performed experiments; C.R., Z.M., T.P., S.H., Z-Q.H., T.R., L.N., R.K., and J.N. analyzed data; C.R., Z.M., S.H., Z-Q.H., T.R., L.N., R.K., C.P.J., and J.N. interpreted results of experiments; C.R., Z.M., S.H., Z-Q.H., L.N., R.K., C.P.J., and J.N. prepared figures; C.R., Z.M., S.H., C.P.J., and J.N. drafted manuscript; C.R., Z.M., T.P., S.H., R.K., C.P.J., and J.N. edited and revised manuscript; C.R., Z.M., T.P., S.H., R.K., C.P.J., and J.N. approved final version of manuscript.
